# Ablation Of Atrial Flutter:Block (Isthmus Conduction) Or Not A Block, That Is The Question?

**Published:** 2002-07-01

**Authors:** Ashish Nabar

## Abstract

It is important to identify residual slow conduction and minimize the chance of resumption of conduction after right atrial isthmus ablation to reduce the chance of recurrence of atrial flutter (AFL). The aim of this article is to discuss the best possible way of confirming a bi-directional isthmus conduction (BIC) block after ablation of an isthmus-dependent AFL. A combination of activation and double potential mapping seems to be the most practical way of acutely confirming the BIC block.

Ablation of a macro-reentrant atrial tachycardia involves: 1) mapping the reentrant circuit to define the conduction zones 2) creating linear lines within the critical zones of slow conduction that extend to anatomical borders and 3) terminating the tachycardia and demonstrating conduction block. The para-tricuspid course of the macro-rentrant circuit of isthmus-dependent atrial flutter (AFL) has been previously well studied [1-5]. The low right atrial isthmus (RAI), lying between the tricuspid annulus (TA), coronary sinus (CS) os and the Eustachian ridge, has been for various functional reasons considered the critical zone of an AFL circuit. These reasons include: 1) induction of AFL following an unidirectional block in the RAI 2) repetitive activation of the RAI by the AFL macro-reentrant circuit 3) slow RAI conduction during AFL (< 0.5m/s) 4) ability to demonstrate concealed entrainment from this region 5) termination of the AFL in the RAI during ablation and 6) non-inducibility of AFL after obtaining a bi-directional isthmus conduction (BIC) block. These observations led Poty et al., to recommend that it was not necessary to induce AFL and that one could directly proceed to isthmus ablation if an AFL with a typical ECG pattern had been clinically documented [6]. The aim of this article is to discuss the best possible way of confirming a BIC block after ablation of an isthmus-dependent AFL. It is important to identify residual slow conduction and minimize the chance of resumption of conduction after successful isthmus ablation. A gap in the ablation line, as small as 0.8 cm, can lead to conduction slowing to 0.3 m/s which may be clinically difficult to detect [7]. Resumption of RAI conduction occurs in 10%-15% of patients, mostly within 30 minutes after the last radiofrequency (RF) application [6-10]. Shah et al., suggest that the chance of lesion recovery is far more common, seen in 50% of the ablated sites, within 1 minute of stopping the RF application [11]. Unfortunately, a sub-acute resumption of conduction within the first 24 hours has also been documented [9]. In follow-up studies performed 1-12 months after flutter ablation, recovery of RAI conduction is seen in 45%-50% of patients, however clinical recurrence of AFL occurs in only 5%-10% of patients during the first year after ablation [12,13]. 

## Activation mapping - mapping around the ablation line

**Figure 1:**
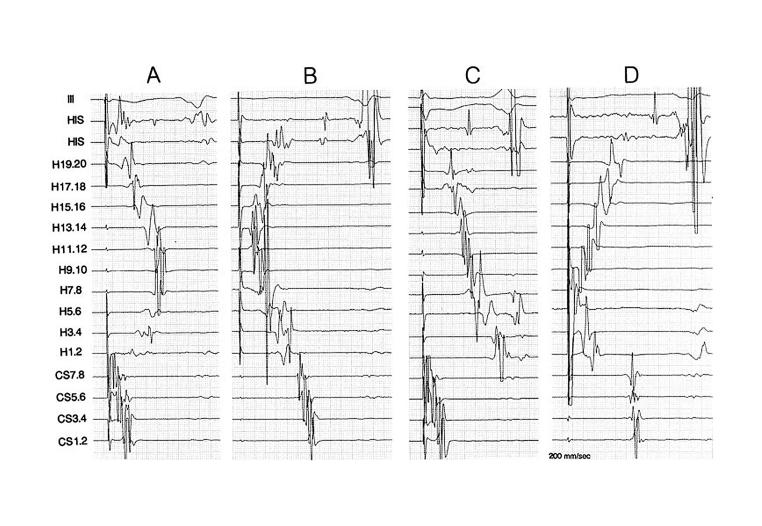
A (pacing at CS orifice) and B (pacing at low lateral RA) represent pacing before ablation to demonstrate BIC. C (pacing at CS orifice) and D (pacing at low lateral RA) show BIC block.

During ablation of an AFL we position a duo-decapolar Halo catheter along the TA, a quadripolar catheter to record His bundle electrogram and a decapolar catheter in the coronary sinus. See Figure 1, panels C and D. A unidirectional right atrial (RA) activation viz. cranio-caudal, along the lateral RA wall during CS pacing and along the septal wall during low lateral RA pacing, after the last RF application had been proposed by Poty et al., as the criteria of BIC block [14]. The assessment of latero-medial isthmus conduction block is to some extent limited by the less extensive mapping of the septum performed using this array of catheters. One has to rely on the activation sequence, activation of the CS os following His bundle activation during low lateral RA pacing. Further, it is often noted that the trans-isthmus intervals prolong gradually until the penultimate application, and jump to their final prolongation after the last RF application [7]. However, this finding might not always be reliably observed in patients who continue anti-arrhythmic drugs at the time of ablation. Actually, the trans-isthmus intervals before and after achievement of BIC block represent activation of different RA structures, and hence by themselves are not meaningful to identify block versus residual slow isthmus conduction. Yet Oral et al., have published that 50% prolongation of trans-isthmus interval is a sure sign of block [15]. An apparent conduction block occurs more often when ablation is performed during AFL than during CS pacing [11]. We demonstrated that the use of isoproterenol infusion, before ending the procedure, could rule out an apparent BIC block [8]. We further stated that pacing close to the ablation line may detect persistent slow conduction. Other way around, in the presence of a BIC block, an "apparent not-block" during CS pacing could be due to conduction behind the inferior vena cava (IVC) along the posterior RA wall, which then penetrates the lower crista terminalis and activates the distal poles of the Halo catheter [16].

## Double potentials - mapping along the ablation line

**Figure 2:**
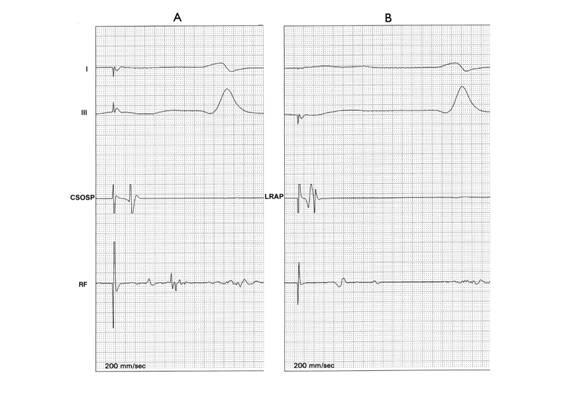
Demonstration of DPs along the ablation line. One catheter is used to pace (A: CS pacing, B: low lateral RA pacing) while the ablation catheter (RF) records along the ablation line.

That double potentials (DP) separated by an iso-electric interval indicate a local block under the recording electrode is known from experimental studies [17]. However, Shah et al., brought this concept to ablation of AFL [18,19]. They have recommended creation of a corridor of DPs, from the TA to the IVC edge of the RAI. This should be verified, both during CS and low lateral RA pacing. See Figure 2. Any single or fractionated (un-seperated) potential along the ablation line, in an acute setting or when re-ablating for an AFL recurrence, should be considered as "gap" and additional RF application delivered. They found that, when activation mapping alone would have suggested a BIC block, in 15% of the patients a persistent conduction was detected by these criteria. Further, conduction along the posterior RA wall, behind the IVC, does not interfere with evaluation of BIC block when using the DP criteria. Another advantage is, essentially the ablation procedure could be simplified to the use of two catheters. Tada et al., attempted to answer, how long the inter-potential intervals should be to discriminate between residual slow conduction and a complete block [20]. According to their calculations, a complete block was associated with an inter-potential interval >110 ms, while an inter-potential interval <90 ms was always associated with residual slow isthmus conduction. When the inter-potential interval was 90-110 ms, an interposed iso-electric segment and a negative polarity of the second component suggested a conduction block. However, it should not be forgotten that the inter-potential interval is dependent on the distance of the recording electrode from the gap, the greater the distance the longer the interval.

## Is any one of the two criteria good enough?

Anselme et al., randomized 76 patients with an isthmus-dependent AFL to ablation using either activation or DP mapping [13]. Activation mapping depends on correct positioning of the Halo catheter, so that the distal bi-poles lie close and anterior to the ablation line. In principle, CS pacing after the last RF application could then rule out any residual slow isthmus conduction. However, correct positioning of the Halo catheter is difficult in 10% of the cases, especially so with the re-use of catheters. DP analysis depends on recording clear-cut widely spaced potentials. Ambiguous potentials are recorded as often as in 40% of the cases.

**Figure 3:**
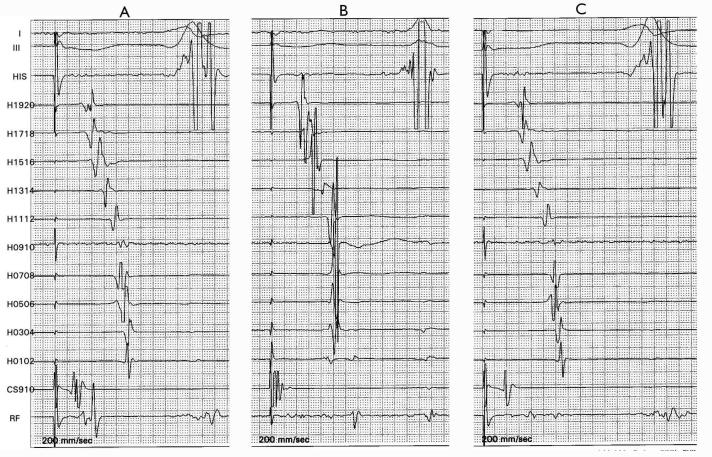
Is any one of the 2 criteria good enough to confirm BIC block? See text for explanations to panel A-C.

See Figure 3, panels A-C. Channel RF represent sites along the ablation line, recorded in different patients after the delivery of the last RF application. Panel A: Halo shows reversal of activation sequence along the lateral RA wall during CS pacing, suggesting an isthmus conduction block. However, a site on the ablation line shows unsplit single potential. Decision: ablate at this site showing a "gap", despite the fact that the Halo catheter suggests (medio-lateral) isthmus conduction block. Panel B: Recordings from the Halo catheter suggests residual slow isthmus conduction. A site with very small amplitude triple potentials was present along the ablation line. These small local potentials are difficult to interpret. What is the significance of a triple potential? Decision: Helped by the fact that recordings from the Halo catheter suggest residual slow conduction, one could infer that the local triple potentials imply slow conduction and ablate at this particular site. Panel C is the most difficult to interpret. The Halo catheter suggests an isthmus conduction block. Locally, a triple potential is noted. Does the central potential suggest slow conduction or bystander activation? After ablation at this site, if the central potential disappears and the interval between the pacing spike and the terminal potential remains same as pre-ablation, it would imply, only retrospectively, that we probably ablated a bystander potential and therefore delivered an unnecessary RF application. However, if the spike to the terminal potential prolongs further, we could say, again only retrospectively, that the RF application was justified and abolished the residual slow conduction.

**Figure 4:**
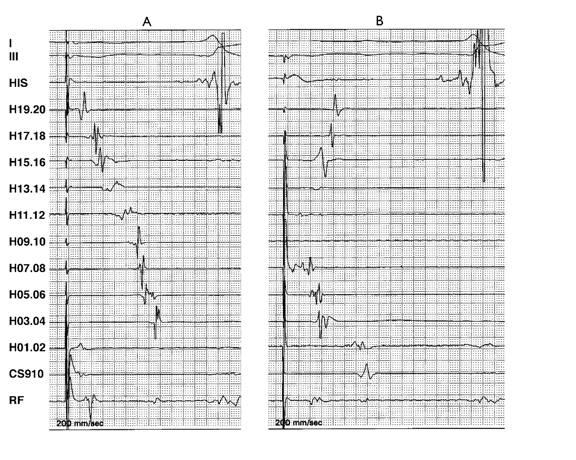
I Concomitant use of activation and local potential mapping to confirm BIC block. A: CS pacing, B: low lateral RA pacing.

Considering these difficulties, Anselme recommended that both methods should be used concomitantly. We routinely use both the methods, as shown in Figure 4, to demonstrate BIC block. CARTO electro-anatomical mapping system, because of its theoretical ability to map unlimited number of points and a lack of restriction regarding mappable sites, often experienced with the use of pre-shaped catheters such as a Halo catheter, could be helpful in difficult cases.

## Other methods to confirm BIC block

Since there is no absolute inter-potential interval that could be considered as a definitive sign of BIC block, maneuvers involving pacing close to the ablation line have been developed by Chen et al [21], and Shah et al [22] Bipolar electrogram polarity reversal to identify a gap in the ablation line has been reported by Yamabe et al, [23] and Tada et al [24] Lastly, the concept that unipolar RS morphology is recorded when the wave front propagates under the recording electrode and a R morphology is recorded at the end of activation has been applied to flutter ablation by Villacastin et al [25] They showed in a group of 45 patients that, after a successful isthmus ablation, a R or an Rs morphology could be recorded in all patients during CS pacing, while a similar morphology can be documented in 90% of patients during low lateral RA pacing. The terminal S remains significant in remaining 10% of the latter patients, probably due to the LA activation which continues to move away from the recording electrode placed posterior to the ablation line.

## Conclusion

We ablate the low RAI during AFL - if this is incessant since the beginning of the study, or during CS pacing - when the rotation of the induced AFL has been studied and this can be readily terminated without the risk of inducing atrial fibrillation. After interruption of the AFL, we continue to ablate the isthmus during CS pacing along the same ablation line. On completion of the ablation line, from TA to the IVC edge, and achievement of a medio-lateral conduction block, as judged by unidirectional cranio-caudal lateral RA wall activation, we perform pacing of the low lateral RA to test for latero-medial conduction block. If this is confirmed, we record local potentials along the ablation line, by withdrawing the ablation catheter in steps, from the TA to the IVC edge while constantly pacing in the CS. If DPs can be demonstrated all along the ablation line during both, CS and low lateral RA pacing, the entire sequence of evaluating BIC block is repeated under isoproterenol infusion. Additional RF pulses are delivered at any sites along the ablation line recording unsplit potentials. Every attempt is made to verify non-inducibility of AFL, unless the risk of inducing atrial fibrillation is very high. Despite this extensive evaluation and the known ability of the RF energy to create full-thickness scar in the isthmus [26], the acute electrophysiological evidence of BIC block may reverse with resolution of inflammation and interstitial edema and may not predict long-term success. Thus the question is not limited to acute confirmation of BIC block after isthmus ablation but extends to: how can we be certain that the conduction block will be maintained over time? Luckily for us, though isthmus conduction may resume the chance of clinical recurrence of AFL seems to be very small.
